# Frequency and factors associated with adherence to and completion of combination antiretroviral therapy for prevention of mother to child transmission in western Kenya

**DOI:** 10.7448/IAS.16.1.17994

**Published:** 2013-01-02

**Authors:** Paul Ayuo, Beverly Musick, Hai Liu, Paula Braitstein, Winstone Nyandiko, Boaz Otieno-Nyunya, Adrian Gardner, Kara Wools-Kaloustian

**Affiliations:** 1 Department of Medicine, School of Medicine, College of Health Sciences, Moi University, Eldoret, Kenya; 2 Department of Biostatistics, Indiana University School of Medicine, Indianapolis, IN, USA; 3 Division of Global Health, Dalla Lana School of Public Health, University of Toronto, Toronto, Canada; 4 Department of Pediatrics, School of Medicine, College of Health Sciences, Moi University, Eldoret, Kenya; 5 Department of Reproductive Health, School of Medicine, College of Health Sciences, Moi University, Eldoret, Kenya; 6 Department of Medicine, Indiana University School of Medicine, Indianapolis, IN, USA

**Keywords:** HIV, pregnancy, combination antiretroviral therapy (CART), prevention of mother-to-child transmission (PMTCT), adherence

## Abstract

**Introduction:**

The objective of this analysis was to identify points of disruption within the prevention of mother-to-child transmission (PMTCT) continuum from combination antiretroviral therapy (CART) initiation until delivery.

**Methods:**

To address this objective, the electronic medical records of all antiretroviral-naïve adult pregnant women who were initiating CART for PMTCT between January 2006 and February 2009 within the Academic Model Providing Access To Healthcare (AMPATH), western Kenya, were reviewed. Outcomes of interest were clinician-initiated change or stop in regimen, disengagement from programme (any, early, late) and self-reported medication adherence. Disengagement was categorized as early disengagement (any interval of greater than 30 days between visits but returning to care prior to delivery) or late disengagement (no visit within 30 days prior to the date of delivery). The association between covariates and the outcomes of interest were assessed using bivariate (Kruskal-Wallis test for continuous variables and the Chi-square test for categorical variables) and multivariate logistic regression analysis.

**Results:**

A total of 4284 antiretroviral-naïve pregnant women initiated CART between January 2006 and February 2009. The majority of women (89%) reported taking all of their medication at every visit. There were 18 (0.4%) deaths reported. Clinicians discontinued CART in 10 patients (0.7%) while 1367 (31.9%) women disengaged from care. Of those disengaging, 404 (29.6%) disengaged early and 963 (70.4%) late. In the multivariate model, the odds of disengagement decreased with increasing age (odds ratio [OR] 0.982; confidence interval [CI] 0.966–0.998) and increasing gestational age at CART initiation (OR 0.925; CI 0.909–0.941). Women receiving care at a district hospital (OR 0.794; CI 0.644–0.980) or tuberculosis medication (OR 0.457; CI 0.202–0.935) were less likely to disengage. The odds of disengagement were higher in married women (OR 1.277; CI 1.034–1.584). The odds of early disengagement decreased with increasing age at CART initiation (OR 0.902; CI 0.881–0.924). The odds of late disengagement decreased with increasing age at CART initiation (OR 0.936; CI 0.917–0.956). While they increased with higher CD4 counts at CART-initiation (OR 1.001; CI 1.000–1001) and in married women (OR 1.297; CI 1.000–1.695)

**Conclusions:**

In a PMTCT programme embedded in an antiretroviral treatment programme with an active outreach department, the majority (67.4%) of women remained engaged and received uninterrupted prenatal CART.

## Introduction

There are 2.3 million children living with HIV globally with almost 90% of them residing in sub-Saharan Africa [1]. In 2009, there were an estimated 370,000 new infections in children acquired from their mother during pregnancy, delivery or through breastfeeding. In the absence of prevention of mother-to-child transmission (PMTCT) of HIV interventions, transmission rates are as high as 40% in sub-Saharan Africa [2,3]. However, PMTCT interventions have reduced transmission to 1.1–6.2% in study and select clinical cohorts in resource-constrained settings [4–11].

The key component of all effective PMTCT strategies is the provision of antiretrovirals to HIV-positive pregnant women [12]. In resource-replete regions, the standard of care is the use of combination antiretroviral therapy (CART) initiated early in pregnancy. However, in resource-constrained settings, various simpler and less costly antiretroviral regimens have been utilized. Historically, single dose nevirapine (sdNVP) prophylaxis given to the mother during labour and to the infant after birth was the most common intervention [13]. However, data indicating the rapid development of NVP-resistant virus in women who receive sdNVP, in addition to studies documenting the benefits of earlier introduction of antiretrovirals as well as the efficacy of CART for prophylaxis has led to the current World Health Organization (WHO)–PMTCT recommendations [5–12][14–17]. For women not requiring CART for their own health, Option A recommends provision of zidovudine (AZT) from 14 weeks of gestation, sdNVP at onset of labour and AZT and lamivudine (3TC) for seven days post-partum with infant prophylaxis during breastfeeding while Option B recommends CART from 14 weeks of gestation with continuation until one week after infant exposure to breast milk has ended [12].

To optimize outcomes from the utilization of these new recommendations, it is necessary to understand and address issues related to programme retention and adherence to antiretrovirals of HIV-positive pregnant women. The data on these issues are limited and difficult to compare because of non-standardized definitions of loss to follow-up (LTFU) as well as varied methods for reporting adherence. For example, data from three studies of care and treatment programmes in South Africa consistently showed that pregnancy was a risk factor for LTFU in patients initiating antiretroviral therapy for treatment [18–20]. However, a multi-country assessment published by the MTCT-Plus Initiative found no difference in retention rates between pregnant women and non-pregnant women or men initiating CART [21]. Within PMTCT programmes offering short course/limited antiretroviral therapy, LTFU rates from antiretroviral initiation to delivery are reported in the range 21.5–68% [22–24]. However, the AMATA study, which initiated CART at 28 weeks, reported 98% retention in the pre-partum period [8]. Factors that have been associated with LTFU include younger age and higher WHO stage. However, the association with CD4 count has been variable with one study identifying no association and another associating LTFU with higher CD4 count [20,21].

In contrast to data from Brazil and the United States, which showed higher rates of adherence in pregnant women, data from sub-Saharan Africa has indicated that there may be significant adherence issues in pregnant women initiating antiretrovirals [25,26]. A Tanzanian study reported full adherence in only 38.4% of women receiving short course antiretrovirals for PMTCT while a South African study (part of the Kesho Bora Study) reported good adherence (>95% adherence) in 61% of their patients with equivalent adherence between women taking CART and those receiving short course antiretrovirals [23,27]. Misconceptions/misunderstandings, antiretroviral use by relatives, domestic violence, poverty and issues relating to disclosure and stigma were reported reasons for the sub-optimal adherence in the South African study, whereas failure to disclose status was the only factor associated with suboptimal adherence in the Tanzanian study [23,27].

The objective of this analysis was to identify points of disruption within the PMTCT continuum from CART initiation until delivery, including lapses in adherence to antiretrovirals, clinician contribution to disruptions, disengagement from care and associated factors. The ultimate goal is to use findings from this analysis to assist in determining points in PMTCT continuum and targeting at risk women for interventions to optimize delivery of PMTCT. This study was conducted within the Academic Model Providing Access to Healthcare (AMPATH) partnership in western Kenya which, from its inception in 2001 to the end of the study period, has used CART-based PMTCT starting at 28 weeks of gestation in women not meeting WHO criteria for treatment [13,28]. Since 2001, the Kenya national guidelines have recommended sdNVP, short course AZT-containing regimens, Option A and Option B, sequentially. The Kenyan Ministry of Health (MOH) is currently considering the adoption of Option B+ (lifelong antiretroviral therapy for all pregnant HIV-positive women)

## Methods

### Study design

This was a retrospective cohort study using routinely collected de-identified data extracted from patient records within the AMPATH medical records system (AMRS) [29]. The Indiana University School of Medicine Institutional Review Board and the Moi University School of Medicine Institutional Review and Ethics Committee approved the study. Institutional review boards approved waiver of consent for this study since it utilized de-identified data abstracted from stored records within an electronic medical record system.

### Study setting

The study was carried out within the AMPATH partnership, which in cooperation with the Ministry of Medical Services and the Ministry of Public Health and Sanitation, delivers HIV care and treatment services to more than 75,000 HIV-positive persons through 65 Ministry of Health facilities in western Kenya [30]. Since AMPATH's inception in 2001, PMTCT has been an integral component of the AMPATH programme.

### Study population

The study population includes all antiretroviral-naïve adult (≥18 years) pregnant women initiating CART from January 2006 to February 2009 within the AMPATH partnership.

### Clinical procedures

Protocols consistent with WHO guidelines for the management of HIV-positive mothers and their children were developed locally and followed for the duration of the study [13]. The 2006 WHO criteria for treatment (all WHO clinical stage 4 patients, stage 3 patients with a CD4 cell count <350 cells/µl and stage 1 and 2 patients with a CD4 cell count <200 cells/µl) were used to determine if a pregnant woman was eligible for treatment for her own health [31]. Women meeting treatment criteria initiated CART on completion of staging while those not meeting criteria initiated CART at 28 weeks of gestation.

Throughout the course of the programme, women who were eligible for therapeutic CART received either AZT or stavudine (D4T) in addition to 3TC and NVP. Women not meeting criteria for therapeutic CART prior to March 2006 received a regimen consisting of either AZT or D4T in addition to 3TC and NVP. After March 2006, patients with CD4 counts greater than 250 cells/µl received nelfinavir (NFV) rather than NVP until July 2007 when lopinavir/ritonavir (LPV) replaced NFV in the PMTCT regimen. During the study period, antiretrovirals were discontinued one to two weeks after delivery in women receiving CART for PMTCT only.

Pregnant women initiating a NVP-based regimen were seen two weeks after initiation and then monthly thereafter while women initiating a protease inhibitor-based regimen were seen monthly (28-day visit schedule). With the exception of the initiation visit for women starting a NVP-based regimen, all women were provided with a 30-day supply of antiretrovirals. During follow-up visits, women were assessed for inter-current symptoms and CART adherence (self-report). Blood pressure, temperature, weight, oxygen saturations and a targeted physical exam as indicated by symptom history were performed at each visit. Per protocol, a complete blood count with white cell differential, CD4 cell count and alanine amino transferase (ALT) were performed at enrolment into the programme and every six months. Additional laboratory testing was carried out based on clinical indications. Viral loads were not routinely collected in this cohort.

Adherence was assessed at every visit using the question; “During the last seven days how many of his/her pills did the patient take?” The available responses were: “none,” “few,” “half,” “most” and “all”. Clinicians document antiretroviral changes or discontinuations at each visit. Available categories for reasons for change/discontinuation include treatment failure, toxicity, completion of PMTCT and other. If a pregnant woman missed a clinic visit by more than seven days and resided in the AMPATH catchment area, an outreach intervention was triggered. Only one contact attempt was made per patient and outreach data was not integrated into the AMPATH database during the period of study. As a result, patients contacted by outreach who did not return to clinic remain disengaged from care from the clinic perspective.

### Review of reason for regimen change

The data on all regimen changes were reviewed by one of the authors (KWK) and grouped into the following categories. *Confirmed toxicity:* the provider indicated toxicity as a reason for change, and per KWK's review the substitution was consistent with toxicity. *Possible toxicity:* the reason for change was given as other or was missing and the antiretroviral change was consistent with substitution(s) routinely made for toxicity. *Nelfinavir purity issue:* In 2007, NFV was pulled from the global market due to concerns about impurities. Single drug changes of NFV occurring during the recall period May 2007–July 2007 were classified as NFV purity issues. *Unknown:* this represents any substitution(s) that is (are) not consistent with a change related to toxicity or NFV purity.

### Data collection and management

The data elements collected on the AMPATH encounter forms at enrolment included demographic, historical, psychosocial, physical exam and laboratory data as well as medications provided (such as antiretrovirals and opportunistic infection prophylaxis). A follow-up encounter form collected data on inter-current symptoms, medication adherence, new diagnoses, laboratory data and additions or changes in drug regimens. Dedicated data entry clerks entered this information into the AMRS [29,30]. Data entry is validated by random review of 10% of the forms entered into the system.

### Analysis

The outcome variables assessed included disengagement from the programme, clinician-initiated regimen change/stop and self-reported medication adherence. Patients were defined as disengaged from the programme prior to delivery if they had no contact with the clinic for more than 30 consecutive days during their pregnancy. “Early disengagement” was defined as any interval of greater than 30 days between visits with a return to care prior to delivery. “Late disengagement” was defined as no visit within 30 days prior to the delivery date (estimated or confirmed). Women who met both definitions were classified as late disengagement. The 30-day window was chosen for this analysis because of the importance of maintaining an 85–95% level of adherence to optimally suppress viral load [32]. Since women were given a 30-day supply of medication at each visit, a gap of more than 30 days represents an opportunity for missed doses of medication. We chose to separate early disengagement from late disengagement because late disengagement may represent a higher potential for an unsuppressed virus at the time of delivery and thus a greater risk of HIV transmission [33].

Perfect adherence to antiretroviral medication was defined as responded “All” to the question “During the last seven days how many of his/her pills did the patient take?” at every visit during pregnancy, whereas imperfect adherence encompassed any response other than “All” at any visit during pregnancy. In this analysis, perfect adherence was not adjusted for the number of recorded adherence measures because the adherence rates are relatively flat across the quantity of adherence measures ranging from 1 to 13 and very few patients had more than ten measures prior to delivery.

Descriptive statistics were generated. Based on the literature and the available programmatic data, an *a priori* decision was made to assess the following covariates for association with the outcomes of interest (perfect adherence; any disengagement, early disengagement and late disengagement): age, gestational age, years of school, CD4 cell count, marital status, employed outside home, WHO stage, clinic type (rural health centre, district (sub-district or district) hospital, referral hospital), clinic location (urban versus rural), concomitant tuberculosis (TB) treatment and components of the CART regimen (AZT versus D4T and a protease inhibitor versus a non-nucleoside reverse transcriptase inhibitor-based regimen) [20,21,23,27,34,35]. We conducted bivariate analysis to explore the associations between the outcome variables and each of the covariates previously listed using Kruskal-Wallis test for continuous variables and Chi-square test for categorical variables. In the multivariate models, all covariates, with the exception of clinic location, were initially included. Clinic location (urban versus rural) is partially co-linear with clinic type, and as such we excluded clinic location from multivariate analysis. We used clinic type in the multivariate analysis because we felt that level of service provision might be a better and more granular factor in predicting retention and adherence than location of the clinic, as this does not necessarily describe the population accessing the clinic or consistently provide information on travel time or cost. For the multivariate analysis, we cycled through covariates in a step-wise fashion and chose the ones that minimized the Akaike information criterion (AIC) to be retained in the final model, which are presented in Tables 2 and 3 [36,37]. Bivariate and multivariate analyses, as described above, were also undertaken to separately explore the covariates associated with early disengagement and late disengagement as compared with engagement. The ten patients whose CART was discontinued by clinicians were not analysed in the multivariate models. All analyses were carried out with R Software (Vienna, Austria) and SAS (version 9.2; SAS Institute, Cary, NC).

## Results

CART was initiated in 4284 antiretroviral-naïve pregnant women between January 2006 and February 2009. Of these, 1298 (30.3%) were pregnant at the time of programme enrolment (Table 1). Most of the women were married (74.4%) and cared for at a district or sub-district hospital (53.0%) rather than at the referral hospital or a rural health centre. The median maternal age at CART initiation was 28.4 (Interquartile range (IQR): 24.1–32.4) years. The median gestational age at CART initiation was 29 (IQR: 28–32) weeks. The majority of women were diagnosed as WHO stage 1 or 2 (79.7%), the median CD4 count was 351 (IQR: 211–511) cells/µl at CART initiation and 18.3% required treatment for their own health.

**Table 1:** Characteristics of women initiating PMTCT within the AMPATH programme (N=4284)

Characteristics	Median	Range	IQR
Maternal age (years) at CART initiation	28.4	18.0–48.2	24.1–32.4
Gestational age (weeks) at CART initiation^a^	29	1–42	28–32
Education (years)^b^	8	0–26	7–11
CD4 Count (cells/µl) at CART initiation^c^	351.0	3.0–2083.0	211.0–511.0
	No.	%	
Pregnant at enrolment	1298	30.3	
Marital status at enrolment			
- Married or living with partner	3189	74.4	
- Never married/not living with partner	488	11.4	
- Divorced/separated	245	5.7	
- Widowed	280	6.5	
- Missing	82	1.9	
Clinic location (urban)	2374	55.4	
Clinic type at CART initiation:			
- Referral hospital	983	23.0	
- District or sub-district hospital	2269	53.0	
- Rural health center	1032	24.1	
Employed outside home			
- Yes	495	11.6	
- No	3701	86.4	
- Missing	88	2.0	
WHO stage at CART initiation			
- 1	2802	65.4	
- 2	613	14.3	
- 3	422	9.9	
- 4	70	1.6	
- Missing	377	8.8	
Met criteria for treatment	784	18.3	
CART regimen:			
- PI-based/AZT/3TC	2688	62.7	
- PI-based/D4T/3TC	476	11.1	
- NVP/AZT/3TC	254	5.9	
- NVP/D4T/3TC	776	18.1	
- Other	22	0.5	
- Missing	68	1.6	
Treatment for TB at CART initiation	89	2.1	

a Number evaluable=a. 3787

b Number evaluable=b. 3944

c Number evaluable=c. 2910.

Of the 3412 women with at least one adherence measure, 3030 (89%) reported taking all of their medication at every visit (Table 2). In the bivariate analysis, imperfect adherence was associated with clinic type, treatment in a rural setting and fewer years of education. Based on the multivariate analysis those receiving treatment at a district/sub-district hospital (odds ratio [OR] 0.681; 95% confidence interval [CI] 0.473–0.964) were 31.9% less likely to report perfect adherence. Each additional year of education (OR 1.106; CI 1.058–1.157) increased the likelihood of reporting perfect adherence by 10.6%.

**Table 2:** Factors associated with CART adherence(perfect versus imperfect) among women enrolling in the AMPATH PMTCT programme from January 2006 to February 2009 (N =3412)

					Bivariate	Multivariate	
					
Factor at CART initiation	Perfect adherence (N=3030)		Imperfect adherence (N=382)		p	Odds ratio (95% CI)	p
Continuous variables; median (IQR)							
Age (per additional year)	28.6 (24.4–32.6)		28.3 (24.1–32.0)		0.328^1^	—	—
Gestation age (per additional week)	28 (28–32)^a^		28 (28–32)^b^		0.834^1^	—	—
Education (per additional year)	8 (7–11)^c^		8 (6–10)^d^		<0.001^1^	1.106 (1.058–1.157)	<0.001
CD4 count (per additional cell/µl)	344.5 (208.0–503.0)^e^		307.0 (173.0–503.5)^f^		0.077^1^	1.000 (1.000–1.001)	0.126
Categorical variables; n/non-missing (%)							
Marital status at enrolment (*married* versus not married)	2222/2966	(74.5)	287/378	(75.9)	0.716^2^	—	—
Employed outside home (*yes* versus no)	380/2961	(12.8)	37/373	(9.9)	0.129^2^	—	—
WHO (*3/4* versus 1/2)	375/3005	(12.4)	53/377	(14.1)	0.431^2^	—	—
Clinic type: *referral hospital*	725/3030	(23.9)	62/382	(16.2)	<0.001^2^	Reference	Reference
Clinic type: District/sub-district hospital	1561/3030	(51.8)	234/382	(61.2)		0.681 (0.473–0.964)	0.034
Clinic type: rural health centre	744/3030	(24.6)	86/382	(22.5)		0.905 (0.590–1.385)	0.646
Clinic location (*urban* vs. rural)	1728/3030	(57.0)	186/382	(48.7)	0.002^2^	NA	NA*
TB Tx (*yes* vs. No)	71/3030	(2.3)	6/382	(1.6)	0.438^2^	—	—
CART regimen (*AZT* vs. D4T)	2086/3017	(69.1)	262/379	(69.1)	0.957^2^	—	—
CART regimen (*PI* vs. NNRTI)	2207/3024	(73.0)	273/379	(72.0)	0.740^2^	—	—

a Number evaluable = a. 2726;

b Number evaluable = b. 333;

c Number evaluable = c. 2793;

d Number evaluable = d.347;

e Number evaluable = e. 2074;

f Number evaluable = f. 268.

1 Kruskal-Wallis.

2 Chi-square test.

* Clinic location (urban vs. rural) is excluded from the multivariate logistic regression model because it provides partial redundant information to clinic type.

—=the corresponding predictor variable was excluded in the multivariate logistic regression model by stepwise search based on AIC.

Italicized factors represent the reference in both bivariate and multivariate analyses.

**Figure 1:**
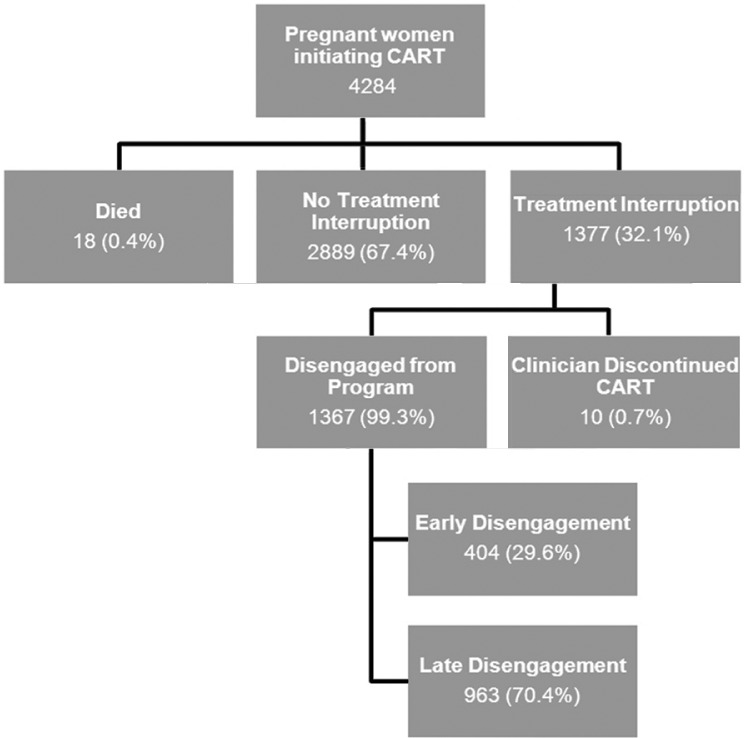
Outcomes of women initiating CART during pregnancy.

Of the women studied, 18 (0.4%) died and 1377 (32.1%) experienced a treatment interruption either due to disengagement or due to clinician discontinuation of CART (Figure 1). Clinicians discontinued CART in only ten patients (0.7%). Clinician reported reasons for CART discontinuation as follows: toxicity 1 (10%), other 6 (60%) and no reason given 3 (30%). Of the 118 women who experienced a regimen change during their pregnancy, the identified reasons included confirmed toxicity 7 (5.9%), possible toxicity 50 (42.4%), NFV purity 59 (50.0%) and unknown 2 (1.7%).

There were 1367 (31.9%) women who disengaged from the programme at some point during their pregnancy. Of these, 404 (29.6%) disengaged early but returned to care prior to delivery (early disengagement) and among the 334 who had information regarding the next scheduled visit, the mean number of days overdue was 15.0 (standard deviation (sd)=17.6). The remaining 963 (70.4%) disengaged from care more than 30 days prior to their date of delivery (late disengagement).

In the bivariate analysis, *any disengagement* (early or late) was associated with younger age, earlier gestational age at CART-initiation, clinic type, and was marginally associated with care in an urban setting (Table 3). In the multivariate model, the odds of disengagement decreased by 1.8% per additional year of age (OR 0.982; CI 0.966–0.998) and 7.5% per additional week of gestational age (OR 0.925; CI 0.909–0.941) at CART initiation. Being married (OR 1.277; CI 1.034–1.584) was associated with increased odds of disengagement. While women receiving care at a district hospital (OR 0.794; CI 0.644–0.980) or tuberculosis medication (OR 0.457; CI 0.202–0.935) were less likely to disengage.

**Table 3:** Factors associated with disengagement among women enrolling in the AMPATH PMTCT programme from January 2006 to February 2009 (N=4256)

			Any disengagement versus engaged				Early disengagement versus engaged				Late disengagement versus engaged		
							
			Bivariate	Multivariate			Bivariate	Multivariate			Bivariate	Multivariate	
							
Factor CART initiation	Engaged (N=2889)	Disengaged any (N=1367)	p	Odds ratio (95% CI)	p	Disengaged EARLY (N=404)	p	Odds ratio (95% CI)	p	Disengaged late (N=963)	p	Odds ratio (95% CI)	p
Continuous variables; Median (IQR)													
Age (per additional year)	28.7 (24.3–32.6)	27.8 (23.8–31.8)	<0.001	0.982 (0.966–0.998)	0.025	28.1 (24.3–31.7)	0.140	0.976 (0.949–1.004)	0.094	27.7 (23.6–31.8)	<0.001	—	—
Gestational age (per additional week)	29 (28–32)^a^	28 (28–30)^b^	<0.001	0.925 (0.909–0.941)	<0.001	28 (26–29)^c^	<0.001	0.902 (0.881–0.924)	<0.001	28 (28–32)^d^	<0.001	0.936 (0.917–0.956)	<0.001
Education (per additional years)	8 (7–11)^e^	8 (7–11)^f^	0.638	—	—	8 (6–11)^g^	0.132	0.956 (0.911–1.003)	0.064	8 (7–11)^h^	0.755	—	—
CD4 count (per additional cell/µl)	350.0 (210.0–513.0)^i^	354.0 (218.5–509.0)^j^	0.714	1.000 (1.000–1.001)	0.067	330.5 (211.0–476.0)^k^	0.240	1.000 (1.000–1.001)	0.139	361.5 (222.0–526.0)^l^	0.239	1.001 (1.000–1.001)	0.029
Categorical variables; n/non-missing observations (%)													
Marital status at enrolment (*married* versus not)	2136/2846 (75.1)	1033/1329 (77.7)	0.065	1.277 (1.034–1.584)	0.025	288/391 (73.7)	0.593	—	—	745/938 (79.4)	0.007	1.297 (1.000–1.695)	0.053
Employed (*yes* versus no)	328/2832 (11.6)	164/1336 (12.3)	0.551	—	—	65/391 (16.6)	0.006	—	—	99/945 (10.5)	0.384	—	—
WHO stage (3 and 4 versus 1&2)	343/2753 (12.5)	141/1132 (12.5)	0.960	—	—	61/399 (15.3)	0.134	—	—	80/733 (10.9)	0.282	0.715 (0.476–1.046)	0.094
Clinic type: *referral hospital*	622/2889 (21.5)	358/1367 (26.2)	<0.001	Reference	Reference	107/404 (26.5)	0.054	—	—	251/963 (26.1)	0.012	Reference	Reference
Clinic type: district hospital	1555/2889 (53.8)	693/1367 (50.7)	<0.001	0.794 (0.644–0.980)	0.031	196/404 (48.5)	0.054	—	—	497/963 (51.6)	0.012	0.788 (0.612–1.018)	0.066
Clinic type: rural health centre	712/2889 (24.6)	316/1367 (23.1)	<0.001	0.817 (0.634–1.052)	0.118	101/404 (25.0)	0.054	—	—	215/963 (22.3)	0.012	0.793 (0.582–1.077)	0.139
Clinic location (*urban* versus rural)	1572/2889 (54.4)	788/1367 (57.6)	0.051	NA	NA*	237/404 (58.7)	0.120	NA	NA	551/963 (57.2)	0.140	NA	NA
TB treatment (*yes* versus no)	67/2889 (2.3)	21/1367 (1.5)	0.119	0.457 (0.202–0.935)	0.043	8/404 (2.0)	0.803	—	—	13/963 (1.3)	0.090	0.436 (0.125–1.172)	0.136
CART regimen (*AZT* versus D4T)	2015/2852 (70.6)	923/1335 (69.1)	0.336	—	—	256/402 (63.7)	0.005	—	0.156	667/933 (71.5)	0.655	—	—
CART regimen (*PI* versus NNRTI)	2174/2860 (76.0)	1000/1336 (74.9)	0.436	—	—	268/402 (66.7)	<0.001	—	—	732/934 (78.4)	0.152	—	—

a Number evaluable = a. 2556;

b Number evaluable = b.1205;

c Number evaluable = c. 363;

d Number evaluable = d. 842;

e Number evaluable = e. 2664;

f Number evaluable = f. 1254;

g Number evaluable = g.369;

h Number evaluable = h. 885;

i Number evaluable = i. 1993;

j Number evaluable = j. 896;

k Number evaluable = k. 270;

l Number evaluable = l. 626.

* Clinic location (rural vs. urban) is excluded from the multivariate logistic regression models because it provides partial redundant information to clinic type.

— = the corresponding covariate variable is excluded in the multivariate logistic regression model by stepwise search based on AIC.

Italicized factors represent the reference in both bivariate and multivariate analyses.

In a bivariate analysis, *early disengagement* was associated with lower gestational age at CART initiation, being employed and a CART regimen containing D4T or a non-nucleoside reverse transcriptase inhibitor (NNRTI). Clinic type was marginally associated with early disengagement (Table 3). In the multivariate model, only gestational age at CART initiation (OR 0.902; CI 0.881–0.924) remained significantly associated, with the odds of early disengagement decreasing by 9.8% per additional week of gestation. In the bivariate analysis, *late disengagement* was associated with younger age, lower gestational age at CART initiation, being married and clinic type. In the multivariate model, gestational age at CART initiation (OR 0.936; CI 0.917–0.956) remained significantly associated, with the odds of late disengagement decreasing by 6.4% per additional week of gestation. With regard to CD4 count at CART initiation (OR 1.001; CI 1.000–1.001), for each additional cell/µl, the odds of disengagement increased by 0.1%. Being married (OR 1.297; CI 1.000–1.695) was identified as being marginally associated with late disengagement.

Of the 2889 women who remained engaged in care throughout their pregnancy, 92.5% had a follow-up visit within 60 days after delivery. The majority (91.6%) of the 404 women with early disengagement adhered to a post-partum clinic visit within 60 days of delivery. Of the 963 women experiencing late disengagement, only 37.5% returned to care within 60 days after they delivered.

## Discussion

In this study, based on very conservative criteria (>30 day gap in care) for disengagement, 31.9% of pregnant women on CART disengaged from care at some point prior to delivery. Of note, 22.5% of all patients were disengaged during the critical time period immediately prior to delivery and only 37.5% of these patients returned to care within 60 days after delivery.

Because of our very conservative definition of disengagement and the short follow-up period required for PMTCT, our findings cannot be directly compared with retention data from our programme or others. However, to provide perspective on the issue of retention, the mean attrition rate (a combination of LTFU and death) within sub-Saharan African HIV programmes has been reported to be 13.9% at six months with death accounting for 41% of the attrition [35]. As noted in the introduction, PMTCT programmes have reported even higher attrition rates (21.5–68%) than ART programmes [22–24]. The relatively high retention rates within our programme may be secondary to a number of structural factors. The presence of an active outreach programme that prioritized pregnant women is likely to have positively impacted retention within our cohort. Unfortunately since outreach records were not part of the AMRS during the study period, we cannot determine the potential magnitude of the impact. Within AMPATH, approximately 36% of women initiating CART for PMTCT were followed by the programme but were ineligible for CART until they became pregnant (internal communication). Given that one-year ART programme attrition rates for ART-ineligible patients are reported to be quite high (24–55.1%), high retention rates in this analysis may reflect survivor bias within the AMPATH programme [38,39]. Lower attrition rates may also reflect a greater sense of engagement with the programme because of their pre-pregnancy connection. There may also be an increased sense of connection to the programme created by receiving CART rather than sdNVP. Though the retention rates are higher than those reported by the majority of PMTCT programmes, we continue to have unacceptably high rates of disengagement during the critical pre- and peri-partum periods, indicating the need to develop interventions to improve engagement of pregnant women by HIV care and treatment programmes.

Increased age, greater gestational age at CART-initiation, receiving TB medications and care at a district hospital were associated with decreased odds of disengagement. Earlier gestational age at CART initiation may be related to higher rates of disengagement simply because of the longer follow-up times necessary for these women. The finding that co-administration of TB medication is inversely related to disengagement was somewhat surprising as we anticipate that these women would have competing health priorities, increased medication-related toxicity, a higher risk of unreported death or hospitalization and as such have higher rates of disengagement. However, it is possible that these women were better engaged by the programme because they have a frequent follow-up for a symptomatic illness that is perceived by the community to require treatment and which is not as stigmatizing as HIV. Further investigation of care structures within the three facility levels is necessary to determine why care at a district hospital might be protective against disengagement.

Married women testing positive for HIV may have fears about disclosing their status to their partners and this may adversely impact retention. HIV counselling and testing for couples as part of antenatal care as well as other efforts to engage males in their partner's antenatal care may address this issue [40]. Similar to Toro's study, we found that late disengagement was associated with higher CD4 counts but not WHO stage at CART initiation [21]. Higher CD4 cell counts are a surrogate for better health status, suggesting that healthier women were more likely to disengage from the programme. This finding is intuitive given that individuals who do not feel ill are less likely to have a strong compulsion to follow-up for health care. To improve engagement, PMTCT programmes may need to target this group for incentives and additional services, including enhanced education about the value of PMTCT.

The reported “perfect” adherence rate (89%) for this cohort is higher than the good/complete adherence rates previously reported from PMTCT populations both in North America and sub-Saharan Africa 38–76% [23,27,41,42]. However, adherence rates reported in this study must be interpreted with caution given that self-reported adherence tends to over-estimate adherence when compared to pill count or pharmacy refill data. Similar to Kirsten's study, we found an association between educational level and adherence [23]. We also found that receiving care at a district hospital was associated with lower levels of reported adherence than care elsewhere. The reason for this finding is unclear and additional studies, using pharmacy refill or pill count data as well qualitative methods, are needed to understand how programme structure and patient factors interact to impede or promote adherence in the PMTCT population.

Only 3% of patients experienced a clinician-initiated stop or change in regimen during their pregnancy. This compares quite favourably with the rate of treatment stop or change in our general patient population (estimated at 8.1% during the first year of treatment) [43]. Of those changing their regimen, the most common reason for regimen changes was related to NFV-purity issues with the second most common reason being toxicity (confirmed or possible). As such, the rate of treatment-limiting toxicity in this population appears to be low at 1.6%. However, we do acknowledge that unmeasured adverse events may be contributing to disengagement from care.

This study is confined to HIV-positive pregnant women who initiate CART. As such, the results cannot be extrapolated to the entire community of HIV-positive pregnant women, some of whom have not accessed testing or treatment services. Based on internal AMPATH monthly reports, the average HIV-testing rates in the antenatal clinic are ≥95% and of those testing positive, ≥95% are successfully referred to the antiretroviral treatment programme. As such, we feel that data from this study can be generalized to HIV-positive women accessing antenatal services in East Africa. During the study period, women not requiring CART for their own health discontinued CART after delivery; as such this study does not address issues of retention and adherence during the breastfeeding periods. Another limitation of this analysis is the use of self-reported adherence data, which are known to overestimate adherence in most populations. In addition, the absence of data for scheduled follow-up visits led to the development and use of a 30-day window between patient visits applied to all patients. This may have led to patients being misclassified as disengaged from care if clinicians extended their follow-up interval beyond the 30-day window. Because the AMPATH clinical guidelines recommend monthly follow-up for pregnant women, it is unlikely that this occurred frequently. Other limitations of this analysis are random misclassification due to clinician error in recording and missing data as evidenced by no reason being given for treatment stop or change. Because patient outreach data were not available within the AMRS during the study period, it is unclear how outreach to patients living in the AMPATH catchment area impacted patient return to clinic and thus it is unclear what biases may have been introduced by this practice into this study. Though it is probable that outreach patients are more likely to return to care, the absence of data on the number of patients outreached in this sample make it impossible to estimate the magnitude of this impact. In addition, we are unable to track whether or not women who disengage from the AMPATH programme have accessed care elsewhere. The strengths of our study include the use of a large patient cohort cared for in both urban and rural settings, allowing these results to be more generalizable to PMTCT programmes in other resource-constrained settings.

## Conclusions

In a PMTCT programme embedded in an antiretroviral treatment programme with an active outreach department, the majority (67.4%) of women remained engaged and received uninterrupted prenatal CART. However, even in this highly integrated programme with active outreach targeting pregnant women, 32.6% of patients had either a gap in care or completely disengaged from care prior to delivery. Future studies are needed to address the impact of male involvement in PMTCT engagement, methods for engaging women in PMTCT who perceive their selves to be healthy and the role of the care delivery structure in maintaining engagement. Interventions that prevent disengagement from PMTCT are necessary to achieve the WHO's goal of zero mother to child HIV transmission.
